# The application of principal component analysis to characterize gait and its association with falls in multiple sclerosis

**DOI:** 10.1038/s41598-021-92353-2

**Published:** 2021-06-17

**Authors:** Andrew S. Monaghan, Jessie M. Huisinga, Daniel S. Peterson

**Affiliations:** 1 College of Health Solutions, Arizona State University, 425 N 5th St., Phoenix, AZ 85282 USA; 2 Department of Physical Therapy and Rehabilitation Science, University of Kansas Medical Center, Kansas City, USA; 3 Phoenix VA Health Care Center, Phoenix, AZ USA

**Keywords:** Neurological disorders, Multiple sclerosis, Risk factors, Predictive markers

## Abstract

People with multiple sclerosis (PwMS) demonstrate gait impairments that are related to falls. However, redundancy exists when reporting gait outcomes. This study aimed to develop an MS-specific model of gait and examine differences between fallers and non-fallers. 122 people with relapsing–remitting MS and 45 controls performed 3 timed up-and-go trials wearing inertial sensors. 21 gait parameters were entered into a principal component analysis (PCA). The PCA-derived gait domains were compared between MS fallers (MS-F) and MS non-fallers (MS-NF) and correlated to cognitive, clinical, and quality-of-life outcomes. Six distinct gait domains were identified: pace, rhythm, variability, asymmetry, anterior–posterior dynamic stability, and medial–lateral dynamic stability, explaining 79.15% of gait variance. PwMS exhibited a slower pace, larger variability, and increased medial–lateral trunk motion compared to controls (p < 0.05). The pace and asymmetry domains were significantly worse (i.e., slower and asymmetrical) in MS-F than MS-NF (p < 0.001 and p = 0.03, respectively). Fear of falling, cognitive performance, and functional mobility were associated with a slower gait (p < 0.05). This study identified a six-component, MS-specific gait model, demonstrating that PwMS, particularly fallers, exhibit deficits in pace and asymmetry. Findings may help reduce redundancy when reporting gait outcomes and inform interventions targeting specific gait domains.

## Introduction

Falls are common in people with multiple sclerosis (PwMS)^1^ and are often caused by altered gait and dynamic instability^2^. Therefore, characterizing gait deficits in PwMS may inform interventions aimed at fall-prevention treatments. Current approaches used to characterize gait^3–6^ vary widely, with little standardization of outcomes or tested domains. Further, identifying distinct gait domains related to falls in MS is challenging, not only because many of the outcomes within and across studies co-vary, but also because falls are complex with multifactorial risk factors including physiological, cognitive, and environmental factors^7^. In older adults^8–10^, Parkinson's disease (PD)^11^, and cognitively impaired^12^ populations, researchers have begun to address this issue by using principal component analyses to identify domains of gait. For example, Verghese et al. identified three orthogonal components of gait in older adults: pace, rhythm, and variability^12^. Lord and colleagues extended this work by expanding the model to include asymmetry and postural control domains^8^.

To date, only one such principal component analysis (PCA) has been performed in PwMS, to the authors' knowledge^13^. This study highlighted a spastic-paretic, ataxic, and unstable gait in MS^13^. However, this was conducted in a small sample with walking was confined to a treadmill. Applying the proposed PCA approach to a larger sample of PwMS can (1) clarify the domains of gait commonly impacted in PwMS and (2) identify which domains are relevant to broad clinical outcomes such as falls, cognitive function, fatigue, and quality of life. Relating specific gait domains to such outcomes may provide early identification for PwMS at risk for, e.g., falls or cognitive deficits. Indeed, more than 50% of PwMS are affected by cognitive impairments and fatigue, such as decrements deficits in executive and attentional functioning. Deficits in these domains can reduce the quality of life and social functioning, and identifying people most likely to experience these changes could facilitate earlier treatment^14–16^. Further, previous PCAs in older adults or MS populations did not incorporate turning, dynamic stability, and lower limb kinematics, components of gait that have been directly related to falls^17,18^.

Therefore, this study has three primary goals: (1) utilize PCA to establish specific domains of gait in PwMS, (2) identify which model-derived domains are different between PwMS who do and do not fall, and (3) relate these domains to relevant outcomes such as cognition, quality of life, and fear of falling, all of which are highly prevalent in MS and linked to fall risk^7^. Creating a more streamlined gait model in MS can (1) reduce redundancy when reporting gait outcomes, (2) provide preliminary evidence to develop and advance clinical gait evaluation through the use of a standardized multi-domain assessment, and (3) provide the basis to advance domain-specific rehabilitation through identification of domain-specific deficits in PwMS.

## Results

### Principal component analysis

**Figure 1:**
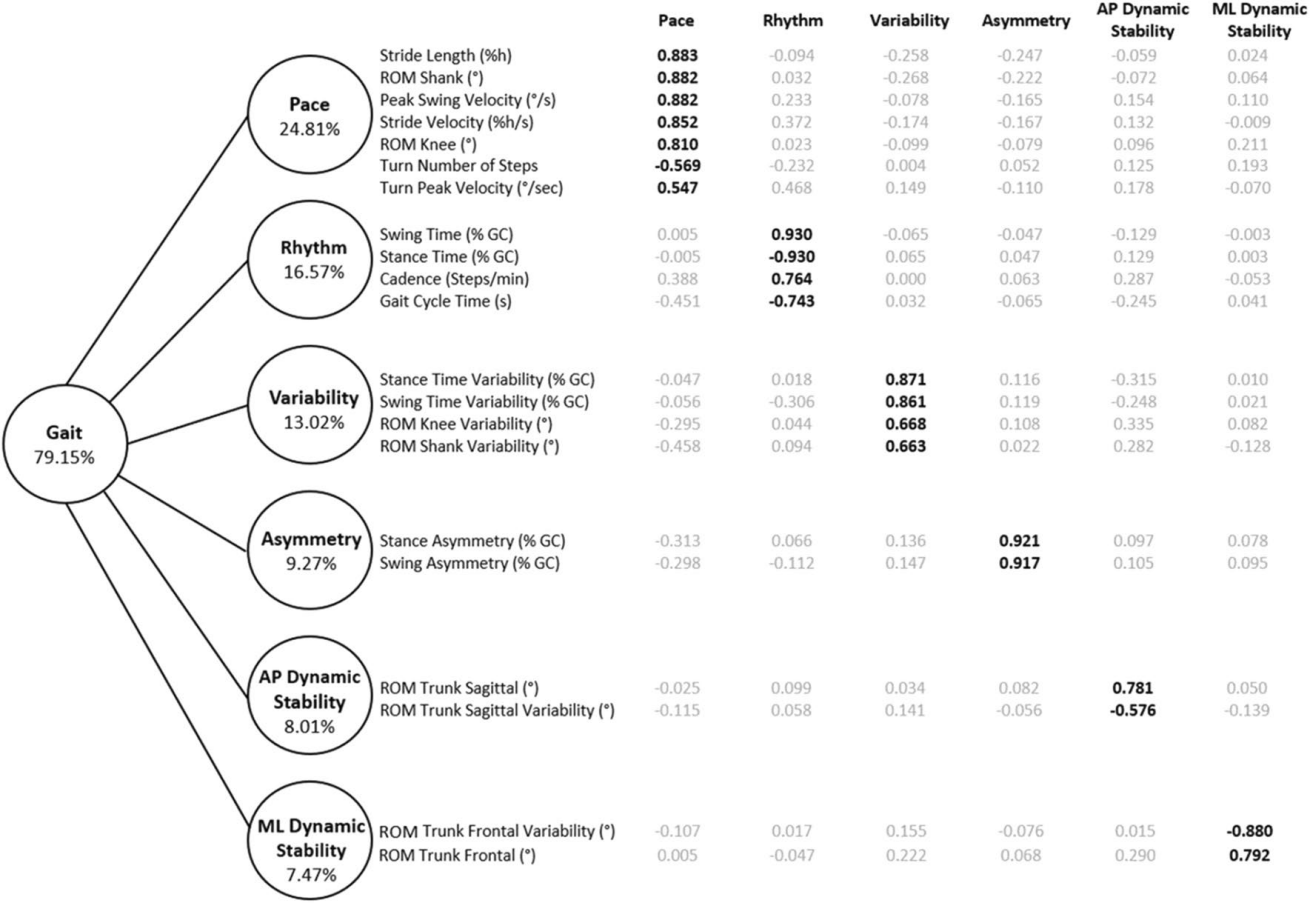
A model of gait for people with MS. A principal component analysis of 21 gait parameters with varimax rotation produced 6 orthogonal domains of gait. Factor loadings were considered relevant at > 0.50 and are bolded. Factor loadings are listed in order of importance. The values in the circles represent the proportion of total variance explained by each domain. *%h* percent of height; *% GC* percent of the gait cycle; *ML* mediolateral, *AP* anterior–posterior; variability was calculated using the coefficient of variation.

Twenty-one gait variables were included in the principal component analysis, yielding six orthogonal components and accounting for 79% of gait variance. The components were labeled as pace (24.81% of total variance), rhythm (16.57%), variability (13.02%), asymmetry (9.27%), anterior–posterior (AP) dynamic stability (8%) and mediolateral (ML) dynamic stability (7.47%). A threshold for relevant item loadings was set at 0.50 or greater, and no cross-loadings were observed. The results of the PCA and factor loadings are shown in Fig. 1.

### Fallers versus non-fallers analysis

**Figure 2:**
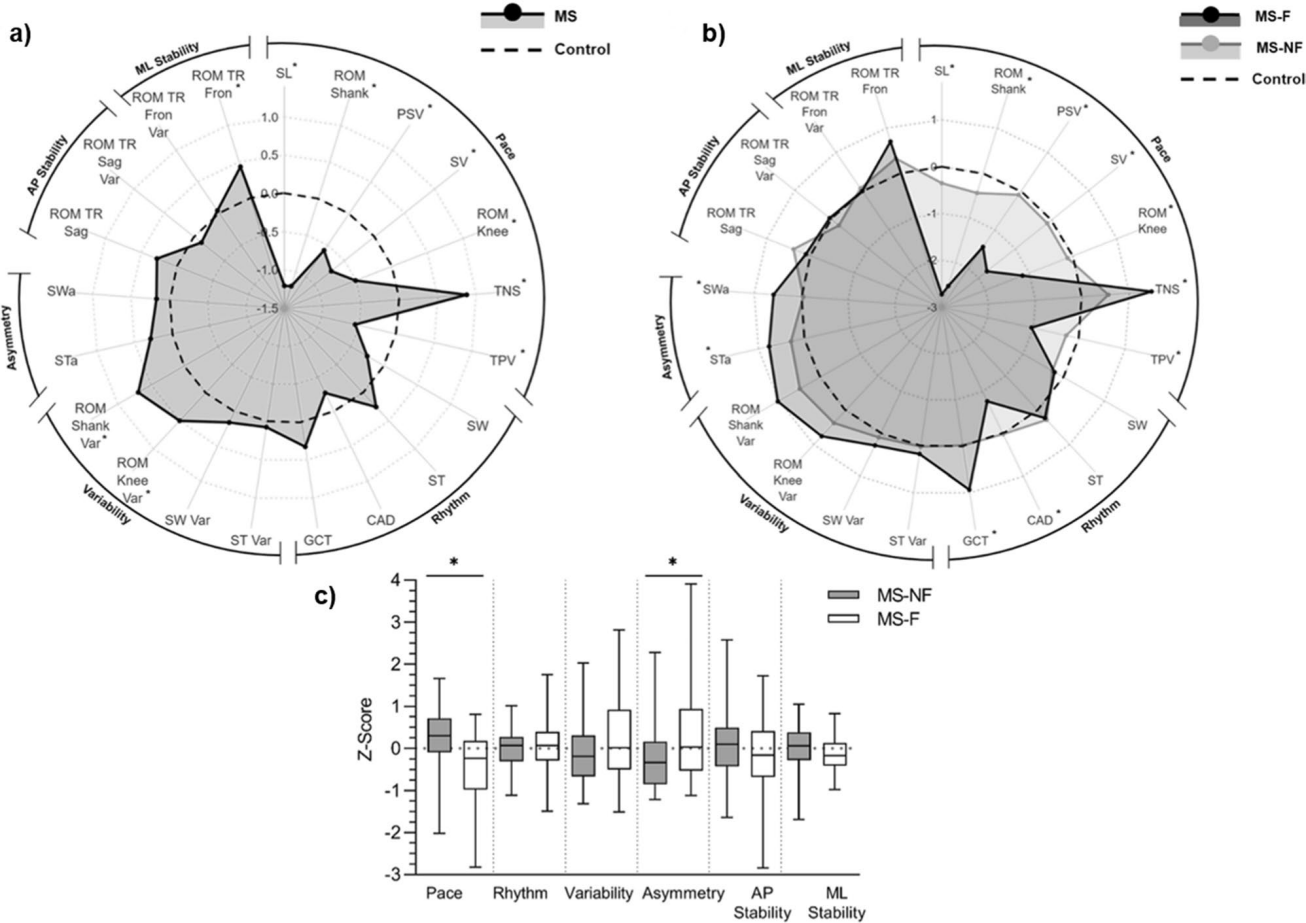
Radar plots illustrating the pattern of gait impairment between PwMS and controls **(a)** and between MS-fallers (MS-F) and MS non-fallers (MS-NF) **(b)**. Data reflect mean values. The central dashed line represents control data. Deviations from zero along the axes indicate the number of standard deviations the MS groups differ from controls. MS gait outcomes were converted to z-scores based on control means and standard deviations. Gait variables are organized by the domains produced in the PCA analysis. **(c)** Represents composite domain scores between MS-F and MS-NF produced by averaging the standardized gait parameters in each domain.* indicates differences between fallers and non-fallers. * indicates a statistical difference) at the p < 0.05 level. *SL* stride length; *ROM Shank* range of motion shank; *PSV* peak swing velocity; *SV* stride velocity; *ROM Knee* range of motion knee; *TNS* turn the number of steps; *TPV* turn peak velocity; *SW* swing time; *ST* stance time; *CAD* cadence; *GCT* gait cycle time; *ST Var* stance time variability; *SW Var* swing time variability; *ROM Knee Var* range of motion knee variability, *ROM Shank Var* range of motion shank variability; *STa* stance time asymmetry; *SWa* swing time asymmetry; *ROM TR Sag* trunk sagittal range of motion; *ROM TR Sag Var* trunk sagittal range of motion variability; *ROM TR Fron* trunk frontal range of motion; *ROM TR Fron Var* trunk frontal range of motion variability.

Seventy-nine PwMS had reported no falls in the last six months, while 42 PwMS had reported at least one fall. MS-F walked with significantly reduced pace compared to MS-NF (p < 0.001, Fig. 2c). The MS-F group walked slower with a reduced swing and stride velocity with short strides than the MS-NF group (p < 0.001 for all; Table 1 and Fig. 2b). Turning metrics were altered across MS-NF and MS-F groups, as MS-F turned slower and took more steps (p < 0.001; Table 1 and Fig. 2b) than MS-NF. Knee and shank range of motion was reduced in MS-F relative to MS-NF. The rhythm domain was also impacted, as MS-F walked with a reduced cadence and prolonged gait cycles than MS-NF (p = 0.003 and p = 0.001 respectively; Table 1 and Fig. 2b). However, this gait domain was not statistically different between MS-F and MS-NF (p = 0.69, Fig. 2c). Finally, MS-F walked with significantly greater asymmetry than non-fallers (p = 0.003; Fig. 2c). MS-F exhibited greater stance and swing asymmetries than MS-NF (both p = 0.01; Table 1 and Fig. 2b). The variability and AP & ML dynamic stability domains were not statistically different between MS-F and MS-NF. Logistic regression analysis showed that the probability of being an MS-F was significantly reduced with increases in the pace domain (β = − 1.347, SE = 0.452, p = 0.003, Exp (B) = 0.260), indicating that a 1 unit increase in pace reduced the odds of being a faller by a factor of 0.26. None of the other gait domains were significant predictors of fall status.

**Table 1:** Mean and standard deviation (SD) of gait parameters in Controls, MS (all), and MS faller and non-faller subgroups.

	Control vs. MS			MS-NF vs. MS-F		
	Control	MS	p-value (Cohen's D)	MS-NF	MS-F	p-value (Cohen's D)
	Mean (SD)	Mean (SD)		Mean (SD)	Mean (SD)	
**Pace**						
Stride length (% height)	88.25 (3.32)	84.25 (9.13)	** < 0.001 (0.50)**	87.08 (6.24)	79.19 (11.31)	** < 0.001 (0.94)**
ROM shank (°)	83.65 (3.60)	79.35 (8.75)	** < 0.001 (0.56)**	82.03 (6.17)	74.56 (10.68)	** < 0.001 (0.93)**
Peak swing velocity (°/s)	430.55 (38.47)	408.1 (58.73)	**0.01 (0.42)**	426.82 (46.84)	375.03 (63.77)	** < 0.001 (0.97)**
Stride velocity (% height/s)	86.86 (7.83)	81.23 (12.72)	** < 0.001 (0.49)**	85.85 (9.95)	73.00 (13.13)	** < 0.001 (1.15)**
ROM knee (°)	58.92 (3.04)	57.38 (4.82)	**0.02 (0.35)**	58.53 (3.63)	55.40 (5.97)	**0.003 (0.68)**
Turn number of steps	3.85 (0.68)	4.45 (0.94)	** < 0.001 (− 0.69)**	4.23 (0.86)	4.86 (0.97)	** < 0.001 (− 0.70)**
Turn peak velocity (°/s)	188.37 (33.98)	169.51 (40.05)	**0.01 (0.49)**	178.67 (40.83)	152.97 (33.35)	** < 0.001 (0.67)**
**Rhythm**						
Stance time (% GC)	61.74 (2.04)	62.26 (2.70)	0.24 (-0.21)	62.31 (2.43)	62.20 (3.20)	0.83 (0.04)
Swing time (% GC)	38.26 (2.04)	37.74 (2.70)	0.24 (0.21)	37.69 (2.43)	37.80 (3.20)	0.83 (− 0.04)
Cadence (steps/min)	118.07 (9.46)	115.47 (11.43)	0.13 (0.24)	118.13 (9.02)	110.78 (13.80)	**0.003 (0.67)**
Gait cycle time (s)	1.02 (0.08)	1.05 (0.11)	0.13 (-0.27)	1.02 (0.08)	1.10 (0.14)	**0.001 (− 0.76)**
**Variability**						
Stance time variability (% GC)	0.02 (0.01)	0.02 (0.01)	0.63 (−0.08)	0.02 (0.01)	0.02 (0.01)	0.23 (−0.23)
Swing time variability (% GC)	0.04 (0.02)	0.04 (0.01)	0.23 (−0.21)	0.04 (0.01)	0.04 (0.01)	0.21 (−0.24)
ROM knee variability (°)	0.03 (0.01)	0.04 (0.01)	**0.01 (−0.48)**	0.04 (0.01)	0.04 (0.02)	0.13 (−0.32)
ROM shank variability (°)	0.02 (0.01)	0.02 (0.01)	**0.01 (−0.42)**	0.02 (0.01)	0.03 (0.01)	0.13 (−0.34)
**Asymmetry**						
Stance time asymmetry (% GC)	2.94 (2.36)	3.61 (3.03)	0.18 (−0.32)	3.03 (2.44)	4.76 (3.66)	**0.01 (−0.59)**
Swing time asymmetry (% GC)	5.30 (4.30)	6.02 (4.88)	0.39 (−0.15)	5.10 (4.19)	7.83 (5.58)	**0.01 (−0.58)**
**AP dynamic stability**						
ROM trunk sagittal (°)	4.36 (1.06)	4.66 (1.43)	0.21 (− 0.22)	4.78 (1.53)	4.47 (1.20)	0.27 (0.21)
ROM trunk sagittal variability (°)	0.18 (0.06)	0.18 (0.05)	0.50 (0.12)	0.17 (0.04)	0.19 (0.06)	0.12 (0.07)
**ML dynamic stability**						
ROM trunk frontal variability (°)	0.19 (0.06)	0.19 (0.06)	0.78 (−0.05)	0.19 (0.06)	0.19 (0.06)	0.71 (0.07)
ROM trunk frontal (°)	8.17 (1.83)	8.97 (2.68)	**0.03 (−0.32)**	8.77 (2.61)	9.46 (2.72)	0.18 (-0.26)

*MS* multiple sclerosis, *MS-NF* people with MS that reported no falls in the last 6 months, *MS-F* people with MS that reported one or more falls in the previous 6 months, *% GC* percent of the gait cycle.

Variability was computed using the coefficient of variation. Bold indicates significance at the p < 0.05 level.

### Regression analysis with clinical outcomes

Pace was the only gait domain significantly associated with clinical outcomes. When controlling for age, disease severity, and duration, increased pace was associated with higher (i.e., better) interference scores on the Stroop task (β = 1.75, SE = 0.81, p = 0.03; Table 2), less fear of falling as measured by the FES (β = − 3.64, SE = 1.07, p =  < 0.001; Table 2), and higher (i.e., better) BBS (β = 0.02, SE = 0.01, p = 0.01; Table 2). There were no significant associations between any clinical characteristics and the rhythm, asymmetry, AP, and ML dynamic stability domains.

**Table 2:** Associations between clinical characteristics and gait domains.

	R^2^	ANOVA *p*	Significant predictor	β (SE)	p value
Stroop	0.15	0.04	Pace	1.75 (0.81)	**0.03**
Fatigue	0.09	0.29	–	–	–
Physical functioning	0.39	< 0.001	Pace	5.98 (3.02)	0.05
Fall efficacy scale	0.37	< 0.001	Pace	− 3.64 (1.07)	** < 0.001**
Berg balance scale	0.43	< 0.001	Pace	2.12 (0.58)	** < 0.001**

Variables controlled for in multiple regressions: age, Expanded Disability Status Scale (EDSS), disease duration, pace, rhythm, variability, asymmetry, AP dynamic stability, ML dynamic stability. Only predictors significant at p < 0.05 level are shown. Coefficients were not interpreted if the ANOVA model was not significant at p < 0.05 level.

## Discussion

This study utilized principal component analysis to create a streamlined MS-specific gait model and directly relate distinct gait domains to falls. The association between gait domains and clinical, cognitive, and quality of life characteristics of MS was also investigated. We identified six distinct gait domains: pace, rhythm, variability, asymmetry, AP, and ML dynamic stability, and the pace and asymmetry domains were worse in MS fallers than non-fallers. Furthermore, pace was associated with subjective fear of falling, cognitive performance, and functional mobility.

The current model of gait in PwMS exhibits subtle differences with PCA analyses performed in other populations. The most recent analyses describe a conceptual model of gait consisting of five domains performed in healthy older adults and people with Parkinson's Disease (PD)^8,9,11^. The pace, rhythm, asymmetry, and variability domains are consistent with prior models. Indeed, many of the loadings are compatible with older adults with the pace domain encompassing stride velocity and length, the component containing the cadence and temporal parameters^9^. The current MS cohort also demonstrated a similar divergence from the healthy older adult gait model seen in PD patients. Specifically, compared to neurotypical adults, both PD and MS models show the gait variability domain to explain more of the total variance than asymmetry than controls. All but two variability measures (frontal and sagittal trunk ROM) were loaded on the variability domain, diverging from the gait model observed in older adults in which variability was dispersed between domains^8^. This may suggest that gait variability is a more salient component of pathological gait.

The additional gait variables in the current study allowed for the expansion of previous models of gait. A postural control domain has been identified in previous analyses and is characterized by step width and step length asymmetry^8,9,11^. However, additional measures of dynamic postural control are needed. The inclusion of trunk kinematics during gait resulted in expanding the previously reported postural control domain^8,9,11^ into two directionally dependent dynamic stability components (AP and ML). Combined, these domains explain approximately 15% of the total gait variance, greater than the ~ 8% explained by the postural control domains in older adults^8^ and PD^11^. The inclusion of trunk kinematics in gait models is warranted, particularly in PwMS, who exhibit a larger trunk range of motion and variability in the ML direction^19^.

Metrics of turning were also a novel inclusion in the current model. Deterioration of balance and coordination have been related to turns and other postural transitions, and the examination of gait beyond straight-line walking is necessary^17,20^. Surprisingly, the inclusion of these parameters did not elicit a turning-specific domain of gait. Instead, both the number of turns and peak turn velocity loaded onto the pace domain. The fact that turning data was averaged from three iTUG trials (each contains a single turn) may have limited the turning metrics' predictive power. Future studies analyses should consider incorporating more turns performed during longer duration walking tests.

Within our cohort of MS participants, we identified disparities in the pace, asymmetry, and, to a smaller degree, rhythm domains of gait between fallers and non-fallers. Specifically, PwMS, who had reported at least one fall in the previous six months, walked significantly slower than those who had not fallen. In fact, all seven gait variables that loaded on the pace domain were statistically different between the two groups (Table 1; Fig. 2). The finding that the pace domain (as defined by the PCA in the MS group) was related to falls reinforces the clinical utility and importance of speed as a measure of walking and lower extremity function in MS. In our study, pace accounted for the most significant variance in gait (24.81%) and (consistent with previous work^21^) discriminated between fallers and non-fallers. Such as finding is significant as walking speed, often measured during the timed 25-foot walk test, is a simple and commonly used assessment both in the clinic and in pharmacological and rehabilitation trials^22^. Therefore, at a time when the clinical evaluation of gait is advancing with technology, the low-tech quantification of pace (e.g., a stopwatch) may provide an effective and inexpensive approach to measure gait, suitable for clinical settings.

In addition to walking slower, fallers also walked with greater stance and swing time asymmetries. Recent investigations suggest that asymmetric gait is a robust measure characterizing the gait in PwMS and related to disease severity and falls^23,24^. Notably, asymmetric gait is not necessarily an uncoordinated gait^25^. Therefore, future studies should include coordination measures such as the phase coordination index^25^, as this measure may also be a relevant outcome for PwMS^23^. Identifying pace and asymmetry as two domains of gait associated with falls is important in assisting with the early prediction of fall risk in MS^26^. It may also support the development of targeted early interventions to decrease the risk of injurious falls in this population.

Surprisingly, variability measures were not statistically significantly different between fallers and non-fallers (p = 0.07). Greater variability has been shown to be predictive of fall status in MS^27^. It is possible that the ability of variability to detect between-group differences was masked due to the short walking assessment. Also, variability is more pronounced and associated with fall risk in moderately-severely impaired PwMS (EDSS score range 4–5)^28^. The MS cohort in this study was less impaired (median EDSS 2.0), which may contribute to the lack of difference in variability metrics between fallers and non-fallers. Finally, despite the lack of difference in gait variability across fallers and non-fallers, the variability domain was related to falls efficacy, underscoring the importance of variability outcomes for PwMS.

Only the pace domain was significantly associated with quality-of-life outcomes. Specifically, and accounting for other domains and possible confounding variables, increased pace was related to lower fear of falling, increased functional mobility, and increased cognitive performance. These findings are partially consistent with previous work. Upwards of 60% of PwMS report a fear of falling, and this outcome has been linked to gait impairments, including lower walking speed and increased stride time variability, and shorter stride lengths^29,30^. A recent meta-analysis showed cognition also to be associated with gait speed in older adults^31^. Specifically, when cognition and gait were portioned into domains, pace was associated with attention and executive function^32^. We observed that increases in pace were related to Stroop interference score improvements, a measure of executive function and attention^33,34^. Significantly, executive function was estimated to correspond to 5- to 10-year deterioration in gait^35^. Together, this work further and specifically links pace with clinically relevant outcomes in PwMS. It further provides preliminary evidence that interventions impacting pace could also, directly or indirectly, improve clinical outcomes such as fear of falling and executive function and attention.

The inclusion of gait data quantified using body-worn wireless inertial sensors is novel to this PCA, as previous studies have utilized some form of a gait mat^8,11^. Inertial sensors enable continuous measurement throughout the entire walking assessment instead of only collecting data while walking on the mat. The use of body-worn sensors enables the measurement of trunk angles, lower limb kinematics, and turning characteristics during gait, in addition to spatiotemporal gait measures. However, spatial data is difficult to capture via body-worn inertial sensors, reducing the ability to capture characteristics such as step length and width via this approach.

There are several limitations to this study. The gait assessment was performed appending three trials of the iTUG protocol. Ideally, gait data, and particularly variability data, would be collected during a continuous walk of a longer duration. The body-worn inertial sensors provide stride characteristics and not step data, so we could not measure step length and step width data (including variability and asymmetry). Also, our MS cohort was restricted to those with relapse-remitting MS and demonstrated relatively minimal impairment (median EDSS 2.0). Therefore, our findings' generalizability is limited to PwMS to MS patients with RMSS and mild severity. Our study accounted for only retrospective falls. It is possible that some participants misestimated the number of falls they experienced in the previous six months, especially given the prevalence of cognitive dysfunction in this population^14^. Future studies should incorporate prospective reporting of falls to avoid such erroneous recall and, when possible, integrate feedback from a spouse or caregiver. In addition, some components of the PCA model may be under-specified (2-items) but were retained as they could discriminate between MS-F and MS-NF. The assessment and interpretation of cognitive function were limited to the domains evaluated in the Stroop Color Word Test. Using the scoring method proposed by Golden & Freshwater 1978^36^, the interference score generated describes the ability to inhibit cognitive interference and fronto-executive functioning^37^. Future studies should examine the association between specific domains and a broader range of cognitive domains such as information processing speed, visuospatial memory, and working memory, which are related to gait in PwMS^38,39^. Finally, while this is a critical first step in creating a more streamlined gait model in MS, PCA analysis only explains communal variance. Therefore, the possibility that variables that are loaded together may not necessarily represent the underlying construct. Future research should conduct confirmatory PCAs in this population to determine the robustness of the domains and constructs identified in the current model.

## Conclusion

This study identified an MS-specific model of gait consisting of six distinct domains. Of these domains, pace and asymmetry were significantly different between MS fallers and non-fallers, and increased pace lowered the likelihood of being an MS-F. The pace domain was also associated with functional mobility and fear of falling. Establishing a more streamlined gait model in PwMS may reduce redundancy in gait outcome reporting for future studies, improve the characterization of clinically observable function and their deficits (i.e., pace, rhythm, timing), and ultimately advance the clinical evaluation and rehabilitation of gait via a standardized multi-domain assessment. Further, identifying which domains are relevant for important outcomes such as falls and fear of falling may assist in the early prediction of fall risk in MS and support earlier interventions to reduce the risk of injurious falls. Future studies should expand analyses to represent a more heterogeneous sample across the MS severity spectrum, and track gait domains' progression and their relation to falls longitudinally. Follow-up work should also investigate the potential imaging markers associated with the distinct gait domains and their association with fall risk. Such investigation would provide insight into the neural correlates of domain-specific gait deficits in MS and may facilitate more targeted rehabilitation.

## Methods

### Participants

A convenience sample of 122 people with relapse-remitting MS and 45 age-matched controls was recruited (Table 3). Participants were recruited via the MS clinic at the University of Kansas Medical Center. Exclusion criteria were: (1) an inability to give consent, (2) an Expanded Disability Status Scale Score > 5.5 or the use of an assistive device, (3) any musculoskeletal or orthopedic impairments that would affect balance or mobility and, (4) any neurological disorder other than MS. All participants provided written informed consent before participation. The study protocol was approved by the ethics board at the University of Kansas Medical Center and was conducted in accordance with the Declaration of Helsinki.

**Table 3:** Participant characteristics. Mean and standard deviation (SD) reported unless noted otherwise.

Measure	MS (n = 122)	CON (n = 45)	
	Mean (SD)	Mean (SD)	p-value
Age	45.51 (9.05)	43.71 (9.54)	0.26
Sex (female/male)	96/26	37/8	–
Height (m)	1.67 (0.10)	1.66 (0.11)	0.70
Weight (kg)	79.5 (20.51)	73.11 (16.14)	0.06
Fallers/non-fallers	42/79	6/39	–
EDSS (range)	2.00 (0.0–5.5)	–	–
Physical functioning (SF-36)	72.89 (24.60)	96.11 (5.32)	** < 0.001**
Energy and fatigue (SF-36)	46.69 (22.12)	70.44 (15.59)	** < 0.001**
Berg balance scale	53.07 (4.84)	–	–
Falls efficacy scale	25.78 (8.51)	18.13 (2.17)	** < 0.001**
Stroop word test (interference score)	20.12 (5.47)	7.34 (3.64)	** < 0.001**

*MS* multiple sclerosis, *CON* control, *EDSS* Expanded Disability Status Scale.

Fall status was determined if one or more fall was reported in the previous 6 months. Berg Balance data was collected in the MS group only. Bold indicates statistical significance at the p < 0.05 level.

### Clinical assessments

The Expanded Disability Status Scale (EDSS)^40^ was used to measure disease severity. Functional mobility was assessed using the Berg Balance Scale (BBS)^41^. The Short Form-36 (SF-36) is a widely used measure of health-related quality of life in PwMS^42^. From the SF-36, we include (1) The Energy and Fatigue subscale to assess fatigue and (2) the Physical Functioning subscale to capture physical health. Subjective fall-risk was obtained via the Falls Efficacy Scale International (FES-I)^43^.

### Cognitive assessments

Executive function was assessed with the Stroop Color and Word Test^36,44,45^. Participants completed three trials in which they (1) read the name of colors [W]), (2) identified the color of rectangles patches [C]), and (3) identified the color of the ink of words with incongruent color-word combinations [CW]. In each condition, participants recited as many responses as possible over thirty seconds. The interference score was calculated as:

$$IG=CW-\frac{\left[W*C\right]}{[W+C]}$$

where IG: interference score. CW, W, and C are the number of correct responses in the CW, W, and C conditions, respectively^44^. Larger IG values reflect better scores.

### Fall assessment

PwMS were classified into two groups; non-fallers (MS-NF) and fallers (MS-F) based on their self-reported fall history in the previous six months. Falls were operationally defined as "An unexpected event in which the participants come to rest on the ground, floor, or lower level”^46^.

### Quantitative gait assessment

#### Protocol

Gait data were computed from the instrumented timed up and go (iTUG) assessment, a reliable and valid measure of gait and turning^6,47^. Participants were instructed to stand up from the chair, walk just past a line placed 7 m straight ahead at a comfortable pace, turn around, walk back, and sit down. The iTUG was extended from the traditional 3 m to enable the computation of gait cycle data. Participants completed three iTUG trials.

### Data analysis

Gait data were collected using Opal wireless inertial sensors (128 Hz). Six sensors placed on the feet, wrists, chest, and lumbar region of the lower back were utilized. Spatiotemporal gait outcomes were determined from inertial measurements and foot positions during the gait cycle measured by the sensors^48^. Specifically, Mobility Lab software (Version 2) (Opal Sensors, APDM Inc., Portland, OR) was used to stream and export gait metrics automatically^48^. This is a reliable and valid system for quantifying gait and mobility dysfunction^49^. The gait cycle's temporal characteristics were computed relative to gait cycle duration, defined as the duration from the foot's initial contact to the next initial contact of the same foot. Steps during gait and turns were detected using the shanks' two sensors^47^. Gyroscopes on the trunk and lumbar sensor detected turns^47^.

### Gait characteristics for principal component analysis

To ensure an adequate number of gait cycles, data from each participant's three iTUG trials were appended. After appending data, the median (range) of gait cycle observations was 20 (12–26) for controls, 21 (11–43) for MS non-fallers, and 22 (15–43) for MS fallers. Mean spatiotemporal parameters, averaged across the right and left limbs, kinematic measures, turning parameters, variability metrics, and asymmetry measures were then assessed. Table 2 outlines gait parameters, and definitions for all gait outcomes are presented in supplementary table 1. The variability of gait outcomes was defined as the coefficient of variation (CoV) computed as standard deviation/mean. Several previous studies have used short walks (i.e., up to 10 m) to report variability in kinematic and spatiotemporal gait parameters^5,50^. Asymmetry across the lower limbs was calculated as gait asymmetry [%] = 100 * |*ln (Right Limb/Left Limb)|*. The number of steps in each turn was measured by taking the average of the three iTUG trials.

Choice of outcomes to include in the model was made to ensure a breadth of spatial and temporal outcomes while limiting redundancy. Specific outcome choice was informed by gait deficits highlighted in MS in systematic reviews and meta-analyses^3,5^, and previous factor analyses^8,9,11,12^. Further, we sought to expand on previous principal component analyses by including metrics of turning and trunk range of motion as a marker of dynamic stability given their importance for walking performance and falls^6,18,51,52^. Variability was defined as the coefficient of variation (CoV) to remain consistent with previous reports^5^.

### Statistical analysis

The data analysis consisted of three parts: (1) a PCA on spatiotemporal gait parameters in MS participants, (2) the comparison and association of gait domains between MS-fallers (MS-F) and non-fallers (MS-NF) using independent t-tests and binary logistic regression, and (3) multiple linear regression analyses of cognitive, clinical, and quality of life characteristics to gait domains. Although not a primary aim of this report, gait parameters were also compared between PwMS and controls. Data were checked for normality using the Shapiro-Wilks test and the visual inspection of histograms. The linearity of variables and normal distribution of residuals was examined via the visual inspection of scatterplots and Q–Q plots for the multiple regression analysis. Homoscedasticity was assessed by visually inspecting a scatterplot of the residuals and predicted values.

A PCA that uses the communal variance of included gait parameters was performed to identify a more parsimonious representation of the latent construct gait in PwMS. Mean gait data from healthy control participants were used for comparative and reference purposes. Therefore, a PCA analysis was not performed with this data. Components were derived using varimax rotation to produce orthogonal partitions. Kaiser's score and Cattell's Scree plot were examined to identify the number of components to extract. Cross-loadings were also examined. Components with a minimum loading of 0.5 were considered relevant. The relationship between fall-status and gait domains in PwMS was tested via independent t-tests and binary logistic regression. Consistent with previous PCA analyses^53,54^, independent t-tests were used to compare the means of gait parameters within each domain across the MS-F and MS-NF groups. To better reflect the unique influence of composite gait domains produced by the PCA model, domain scores were computed and analyzed. Composite domain scores were attained using an approach described in Maidan et al., 2021^55^. All gait parameters were normalized by subtracting their means and dividing by the standard deviation, followed by the averaging of the normalized measures within each domain identified by the PCA model. For example, the asymmetry domain score was computed by averaging the normalized swing and stance asymmetry measures. The composite domain scores were compared across groups using independent sample t-tests. Logistic regression was also performed to determine if specific gait domains were related to the likelihood of being classified as an MS-F or MS-NF. The dependent variable was coded as 1 = faller, and 0 = non-faller. The predictors were the 6 domain scores and control variables including age, expanded disability status scale (EDSS), and disease duration.

The gait domain scores were also related to clinical, cognitive, and quality of life cognitive characteristics using a multiple regression model. Each measure was entered into the regression model as the dependent variable, with the gait domains as the independent variables. Dependent measures were cognition (interference score of the Stroop Word Test), fatigue (Energy and Fatigue subscale of the SF-36 Scale), physical functioning (Physical Functioning subscale of the SF36-Scale), fear of falling (FES-I), and functional mobility (BBS).

## Supplementary Information

Supplementary Table 1.

## Data Availability

All data are available following reasonable request.
