# Supplementary material for: The application of principal component analysis to characterize gait and its association with falls in multiple sclerosis
Source: Sci Rep. 2021 Jun 17;11:12811. doi: 10.1038/s41598-021-92353-2 (PMC8211858; doi:10.1038/s41598-021-92353-2)
Supplement: Supplementary file 1 — Supplementary Table 1. [file 41598_2021_92353_MOESM1_ESM.docx]

| **Supplementary Table 1** Operational definitions of gait characteristics included in the factor analysis | |
| --- | --- |
| **Gait Outcome** | **Definition** |
| **Spatiotemporal Measures** |  |
| Stride Velocity (% height/s) | Average gait speed normalized for height |
| Stride Length (% height) | Distance between two consecutive heel strikes normalized for height |
| Stance Time (% Gait Cycle) | The average percentage of a gait cycle that either foot is on the ground |
| Swing Time (% Gait Cycle) | The average percentage of a gait cycle that either foot is off the ground |
| Cadence (steps/min) | Stepping Rate |
| Gait Cycle Time (s) | Duration of a complete gait cycle |
| **Turning Measures** |  |
| Turn Peak Velocity (°/sec) | Peak (95%) angular velocity of the trunk during turning |
| Turn Number of Steps | Total number of steps during a 180-degree turn |
| **Dynamic Stability Measures** |  |
| ROM Trunk Sagittal (°) | The average range of motion of the trunk in the sagittal plane |
| ROM Trunk Frontal (°) | The average range of motion of the trunk in the frontal plane |
| **Joint Kinematics** |  |
| Peak Swing Velocity (°/s) | Peak (95%) angular velocity of the swing leg |
| ROM Shank (°) | Range of motion of the leg (calculated from the integrated sagittal angular velocity, approximation of step length). Average of the left and right sides. |
| ROM Knee (°) | Range of motion of the knee (calculated from the integrated sagittal angular velocity, approximation of step length). Average of the left and right sides |
| **Variability Measures** | The standard deviation of the gait outcome compared to the mean (coefficient of variation). Computed using the formula: (SD/mean) |
| Stance Time Variability (% Gait Cycle) |  |
| Swing Time Variability (% Gait Cycle) |  |
| ROM Knee Variability (°) |  |
| ROM Shank Variability (°) |  |
| ROM Trunk Sagittal Variability (°) |  |
| ROM Trunk Frontal Variability (°) |  |
| **Asymmetry Measures** |  |
| Swing Time Asymmetry (% Gait Cycle) | The comparison of the right swing time to the left swing time using the formula: *'100 * \|ln (Right Swing Time / Left Swing Time\|* |
| Stance Time Asymmetry (% Gait Cycle) | The comparison of the right stance time to the left stance time using the formula: *'100 * \|ln (Right Stance Time / Left Stance Time\|* |
